# Incorporating Evidence-Based Gamification and Machine Learning to Assess Preschool Executive Function: A Feasibility Study

**DOI:** 10.3390/brainsci14050451

**Published:** 2024-04-30

**Authors:** Cassondra M. Eng, Aria Tsegai-Moore, Anna V. Fisher

**Affiliations:** 1 Department of Psychiatry & Behavioral Sciences, Stanford University, 1520 Page Mill Road Stanford, Stanford, CA 94304, USA; 2 Department of Psychology, Carnegie Mellon University, 5000 Forbes Avenue, 335I Baker Hall, Pittsburgh, PA 15213, USA; fisher49@andrew.cmu.edu; 3 Department of Psychology, Columbia University, 406 Schermerhorn Hall, 1190 Amsterdam Avenue, New York, NY 10027, USA; ast2195@columbia.edu

**Keywords:** executive function, gamification, assessment, flanker, machine learning

## Abstract

Computerized assessments and digital games have become more prevalent in childhood, necessitating a systematic investigation of the effects of gamified executive function assessments on performance and engagement. This study examined the feasibility of incorporating gamification and a machine learning algorithm that adapts task difficulty to individual children’s performance into a traditional executive function task (i.e., Flanker Task) with children ages 3–5. The results demonstrated that performance on a gamified version of the Flanker Task was associated with performance on the traditional version of the task and standardized academic achievement outcomes. Furthermore, gamification grounded in learning science and developmental psychology theories applied to a traditional executive function measure increased children’s task enjoyment while preserving psychometric properties of the Flanker Task. Overall, this feasibility study indicates that gamification and adaptive machine learning algorithms can be successfully incorporated into executive function assessments with young children to increase enjoyment and reduce data loss with developmentally appropriate and intentional practices.

## 1. Introduction

Executive function (EF) is an umbrella term for cognitive processes that support adaptive goal-directed behavior in the face of changing task demands [1,2]. While there is no consensus on a standard definition of EF, many frameworks posit that EF comprises three components: working memory (the ability to actively maintain and update task-relevant information), cognitive flexibility (the ability to adapt goal-directed behavior in response changes in the environment), and inhibitory control (the ability to override prepotent responses) [3,4,5,6,7,8]. There is no consensus whether EF components are rooted in a common mechanism or reflect different underlying mechanisms, particularly based on the developmental periods being studied [3,9,10,11,12,13]. Furthermore, it is widely recognized that many EF assessments engage multiple components, the so-called task impurity problem [11]. Nevertheless, there is broad agreement that EF plays an important role in higher level cognitive abilities such as planning, reasoning, and problem-solving; develops throughout childhood and adolescence with continued refinement into adulthood; and plays a crucial role in supporting academic achievement, interpersonal and occupational success, and overall wellbeing [14,15,16,17].

Evaluating EF in childhood can be challenging due to children’s limited attention spans and low tolerance for boredom. Therefore, a number of EF assessments have been specifically designed to be developmentally appropriate and developmentally sensitive for use with young children (for review, see [18]). These assessments commonly incorporate game-like features to maintain task engagement. For example, McClelland and colleagues developed [19,20] and revised the [21] Head-Toes-Knees-Shoulders (HTKS-R) task to assess EF through a game-like Simon-Says structure. Specifically, in this task an experimenter instructs a child to “do the opposite” when asked to follow simple prompts such as touching one’s toes when told “touch your head” or touching one’s knees when told “touch your shoulders”. New rules can be added to increase task difficulty and a condition was added in the revised version for children to “say the opposite” first to decrease difficulty. This task engages multiple EF components as successful performance requires children to suppress prepotent motor responses (inhibitory control), switch responses when rules change (cognitive flexibility), and actively represent current rules (working memory) [21]. Other similar EF tasks for children involve verbal responses, including the Day-Night, Mommy-Me, Yes-No, and Grass-Snow Tasks [5,18,22,23]. For example, in the Day-Night task, children are first instructed to say “Day” for cards showing a Sun image and say “Night” for cards showing a Moon image, thereby activating established connections between the words and their corresponding images for congruent trials. Then, the rule switches and children are instructed to say “Night” for cards showing a Sun image and “Day” for cards showing a Moon image for incongruent trials. Task difficulty can be increased by introducing new rules (e.g., children may be instructed to give a congruent or incongruent response based on the border color of the stimulus card) [24]. Similar to the HTKS-R task, the Day-Night task recruits multiple EF processes as children need to maintain current task goals in mind (working memory), suppress a practiced response to provide a conflicting response on incongruent trials (inhibitory control), and adapt to switch responses when the rule changes (cognitive flexibility).

The tasks briefly described above have been widely used to assess EF in young children. However, administering these tasks can be resource intensive as trained experimenters need to manually code behavioral response accuracy and keep track of rule changes as the task increases in complexity. Furthermore, manual scoring of performance can lead to observer bias and error. These challenges can be solved through the use of computerized assessments, and some computerized tasks developed for adults have been adapted for use with children. One common computerized EF assessment that was adapted for use with children is the Go/No-Go task, in which children need to execute a motor response (e.g., button press) as fast as possible in response to some stimuli (i.e., Targets) and withhold response to other stimuli (e.g., Non-Targets) [25]. The common distribution of stimuli in this task includes 80% Targets (i.e., ‘go’ stimuli) and 20% Non-Targets (i.e., ‘no-go’ stimuli), thus rendering ‘go’ the prepotent response. This task requires participants to actively maintain rule representations of Target and Non-Target stimuli (working memory), and inhibit prepotent ‘go’ responses. In contrast, in the Continuous Performance Task (CPT), another computerized EF measure, participants monitor for infrequently appearing Targets among frequent Non-Target stimuli, thus having to initiate infrequent response (e.g., button press) while withholding a motor response most of the time. Both the Go/No-Go and CPT tasks were first developed for use with adults and then adapted for use with children by replacing letter or number stimuli (often used with adults) with pictorial stimuli, increasing inter-stimulus intervals, and decreasing the overall task length (e.g., [26,27]).

Computerized EF assessments for children are widespread in the literature [28]; however, these assessments have complementary strengths and weaknesses when compared to the non-computerized assessments. On the one hand, computerized assessments reduce concerns about observer bias and human error in data coding, allow more fine-grained EF assessment by enabling measurement of not only response accuracy but also response latency, and reduce demands on experimenters to keep track of rule changes (often used to increase task difficulty to avoid ceiling effects). On the other hand, children often struggle to maintain task engagement in computerized assessments, which can lead to substantial data loss. For example, despite child-friendly adaptations to the CPT task, nearly 50% of participants below 4.5 years of age do not provide enough usable data for inclusion in data analyses (for review see [29]).

A potential solution that can reduce challenges posed by computerized assessments while preserving their benefits is gamification of an EF task as a Gamified Assessment (GA): the addition of game features to computerized task-based measures to advance psychometric measurements [30]. GAs potentially possess the capability to engage children in a well-established EF task in an ecologically valid context with increasing challenge without adding a separate condition. GAs can incorporate algorithms to dynamically adjust the difficulty level based on individual performance, ensuring that children are appropriately challenged and avoiding the floor or ceiling effects commonly seen when a study involves participants of varying ages. There is recent evidence that suggests GAs offer high test sensitivity for evaluating EF in school-age children [31]. Furthermore, developing valid and reliable GAs of EF in a digital format that children commonly encounter in their everyday educational and home environments might be an initial method in addressing concerns about the ecological validity of task-based EF assessments conducted in laboratory settings.

Methods of assessment adapt as new generations emerge in continually evolving developmental contexts. Due to the complex and rapid development of EF in the preschool years—coupled with the growing use of technology among youth in the digital age—there is considerable interest in exploring the optimal computerized EF assessments that are feasible, scalable, and enjoyable for children. The wide-spread implementation of remote data collection, telehealth assessments, and gamification incorporated into K–12 education curricula bring forward the need to investigate the effects of gamification in computerized childhood EF assessments [32]. This area is timely because digitized EF assessments are growing rapidly due to increased access to mobile devices, standardization that reduces human labor and error, adaptability to diverse cognitive profiles, automatized data collection and storage, and features to increase children’s engagement. Children under the age of 8, on average, play digital games for 23 min daily, 98% have access to a mobile device at home [33], and 95% of K–12 teachers report using digital tools in classrooms [34] in the United States. Thus, assessing EF through computerized GAs may be ecologically valid because children are exposed to a variety of digital formats and games in the classroom and at home.

Gamification of assessments has been shown to be especially beneficial for individuals for whom traditional approaches are often unsuccessful, likely reflecting the sustained engagement and motivation that gamified tasks evoke [35]. For example, prior work shows that adding game features and machine learning—the use of participant data in real-time and algorithms to gradually adjust difficulty level to challenge participants appropriately—to the CPT have been especially beneficial for the engagement of individuals with EF impairments such as those with ADHD [36,37]. Similarly, Ahufinger and Herrero-Martín [38] found that participants with ADHD ages 6–13 completed GAs of EF under the time allocated, with feedback from participants that they would have preferred increased task difficulty. Additionally, GAs of EF assessments can also be highly useful for research with neurotypical samples: typically developing participants without psychiatric disorders often show performance in the relatively narrow ranges in traditional EF assessments [39], and GAs permit a more nuanced measurement of performance. Finally, prior studies report that adult participants prefer GAs to traditional EF assessments [40]; and participant preferences for more enjoyable tasks can be an important factor in task compliance and completion, especially with child participants.

The present study reports the feasibility of adapting the Eriksen Flanker Task [41], a measure foundational in understanding EF related to individual differences, brain-behavior associations, and psychiatric disorders [42], as a gamified EF assessment. In the Flanker task, participants are instructed to press a button matching the direction of a central target arrow surrounded (flanked) by peripheral arrows [43]. On congruent trials, the surrounding arrows point in the same direction as the central target; and on incongruent trials, the surrounding arrows point in the opposite direction compared to the central target. Therefore, participants need to narrowly focus attention on the central target while inhibiting distraction from the flanking arrows surrounding the target [44]. The Flanker Task engages EF by requiring participants to override the prepotent action to respond in the direction of the distractor flankers (inhibitory control), adapt responses when the target switches directions and the task dimensions change between congruent and incongruent trials (cognitive flexibility), and maintain the task goal of responding to the central target while simultaneously monitoring competing demands of ignoring the distractors (working memory) [45].

Rueda and colleagues [46] developed the first modified version of the Flanker Task for use with children and replaced arrows with pictures of fish as stimuli. Similar to the adult version, trials consist of a horizontal array of five arrows, and children are instructed to respond to the direction of the central fish and ignore the distracting flanking fish. McDermott, Perez-Edgar, and Fox [47] conducted a study investigating variations of the flanker task and found that children exhibit the greatest conflict scores with fish as stimuli in comparison with color and shape versions of the Flanker Task. The researchers suggest that the strategic use of directionality knowledge creates stronger response competition during incongruent trials in the fish version: children’s EF skills are being taxed more strongly by the incompatible stimuli that automatically and simultaneously activate an alternative response due to prior knowledge of directionality.

Rueda and colleagues [46] reported no data loss in a study with children ages 6–10. However, other researchers using this child-friendly adaptation of the Flanker Task reported data loss when testing younger children. For example, McDermott et al. [47] reported that 11% of participants 4–6 years of age had to be excluded. Becker and colleagues (2023) found 30% of participants 3 years of age failed to pass the practice trials on the standardized NIH Toolbox Flanker Task and 70% performed at or below chance level [48]. Simmering et al. ([49]; Exp. 1a) reported that 30% of participants 3–5 years of age had to be excluded from analyses of Flanker Task data. Simmering and colleagues [49] found that young children exhibit poor performance (high error rates and slow RTs) and difficulty remaining engaged enough to complete the Flanker Task, leading to high data exclusion. Simmering et al. [49] were able to reduce the rate of data loss to below 10% by making significant modifications to the task, including replacing detection of conflict in the direction of a target stimulus in an array with detection of color mismatch between the target and flanking stimuli. However, this new condition exhibits several distinctions from the traditional version of the Flanker Task utilized with older children and adults, thus making direct comparisons with older participants challenging.

Prior research shows promising results on the validity and engagement from the incorporation of gamification into EF assessments with neurodiverse populations, older children, and adults. For example, gamification of the Flanker Task—with the addition of reward incentives and an adaptive algorithm so the response duration shortens with correct responses and lengthens with incorrect responses—has been validated with young adults and elderly populations [50]. However, the feasibility of gamified computerized EF assessments with preschool children is understudied. We report here the outcomes of a feasibility study aimed at filling this gap.

The present study used evidence-based principles of gamification associated with motivation and learning to gamify the child-friendly version of the Flanker Task commonly used to assess EF in children. The implementation of gamification is a redesign strategy employing concepts from game design to existing assessment methods [51]. Following a game mechanic and developmental psychology framework, the success of gamification into traditional assessments depends on effectively integrating features focusing on population appropriate and intentional practices [51]. Thus, the game features employed in the current study are grounded in evidence-based approaches in the learning sciences and developmental psychology that provide opportunities for growth through incremental challenge, appropriate feedback, and harness the motivational nature of games [40,52]. Specifically, we incorporated a storyline that assists participants in envisioning themselves on a quest, player feedback (including both positive incentives and anticipation of an opponent’s behavior), and a staircase machine learning algorithm to offer gradual challenges by dynamically adjusting the difficulty level according to real-time performance, mimicking scaffolding [53,54,55]. In adult–child interactions, scaffolding occurs when adults appropriately adjust the level of support to match the child’s level of performance; thus, the level and type of adult scaffolding is reciprocal to the child’s development, and the interchange between the two is a dynamically calibrated process [52]. Vygotsky suggests that children’s learning and development are optimized through scaffolding and are best facilitated by progressively more complex challenges within the zone of proximal development: the distance between the actual developmental level as determined by independent learning and the level of potential development [56]. The zone of proximal development is found by matching scaffolding to the perceived or actual difficulty experienced by children, which encourages children to engage in progressively more complex patterns of learning.

GAs are capable of providing scaffolding through incremental challenge with appropriate support through the inclusion of (1) algorithms for continuously adapting the difficulty level based on real-time individual performance such as adults do in quality adult-children interactions to keep children consistently in the zone of proximal development making the task neither too easy nor too difficult for children’s level of development [57] and (2) evidence-based game features known to increase engagement including a narrative, an incentive system that takes into account natural desire for competition and rewards, and immediate visual input on performance that provides children with appropriate feedback on their progress [40]. This study explored the impact of a gamified EF assessment with the incorporation of evidence-based features on engagement and desire of continued participation [52].

Given the limited research on the feasibility of GAs of EF in research and clinical settings with preschool-aged children, the goals of this study were as follows. First, we aimed to conduct a preliminary psychometric study of a gamified Flanker Task with children ages 3–5. Specifically, we examined whether the addition of evidence-based game features to the Flanker Task reproduces the well-established conflict effect (i.e., more accurate and faster responses on congruent compared to incongruent trials) in task performance. Second, we report the association between task performance and standardized academic achievement measures observed in prior research. Finally, we assess the effects that gamifying the Flanker Task has on children’s enjoyment and activity preference.

## 2. Materials and Methods

This is a feasibility study, which involves conducting a preliminary experiment with a limited sample size to assess the effectiveness and practicality of research methods, materials, and procedures to be implemented on a larger scale. Twenty participants ages 3 to 5 (*M* = 4.76, SD = 0.97; 7 Females; 13 Males) were recruited from a preschool in Pittsburgh, Pennsylvania, in the United States. One participant was excluded due to noncompliance on the Flanker Task. The school environment represents local racial and economic diversity with children being 54% White, 24% Asian or Pacific Islander, 5% African American, 12% Middle-Eastern, 5% Hispanic, and 28% of children attending with financial aid. The study was approved by the University Institutional Review Board. Signed consent was obtained from the parents of participants. Children were given a small prize for their participation.

For this study, a within-subjects design was implemented in which children participated in two conditions of an EF assessment: the Flanker Task (traditional) Condition and a novel GA Flanker Condition: *Frankie’s Big Adventure* (described in detail below in Section 2.2). To account for order, practice, and fatigue effects, the sequence in which each Condition was played was counterbalanced. Following participation in each Condition, enjoyment levels were assessed. After participating in both Conditions, children were asked which activity they would play again. During subsequent lab visits within the same week, performance on standardized assessments of Verbal and Mathematical tests were collected. Testing sessions were administered to participants in the same room each day, by experimenters naive to the study hypotheses.

The traditional Flanker Task and GA were presented on a MacBook Pro 13″ with a 13.3″ diagonal Retina display with a resolution of 2560 × 1600 with a connected keyboard. The Flanker Task and GA were programmed into an App using Unity Technologies (Version 2019.4.10): software permitting game customization to carefully control features (see Table 1 for similarities and differences between the two conditions). This method permitted programming the novel GA and traditional version of the EF task to be presented on the same software platform, identical in function, response keys, and developed to produce the same output measures [58]. See Supplementary Materials for example videos of the Flanker Task and GA interfaces.

For both Conditions, children completed 50 trials, 8 practice trials and 42 test trials, responding using the left and right buttons on the keyboard. Children completed 8 practice trials to ensure directions were understood, followed by 42 test trials. Practice consisted of intermixed congruent and incongruent trials (four each) with feedback. If participants did not clear the 8 practice trials, the experimenter reiterated the directions and had children practice again to ensure the rules were understood. During the practice trials, children were encouraged to respond as quickly and accurately as possible. No encouragement or correction was given during the testing block. Approximately a 67:33 ratio of congruent trials and incongruent trials was used for both Conditions. Mean accuracy and reaction time (RT) for congruent and incongruent trials, conflict scores (the difference between congruent and incongruent performance), and enjoyment outcomes were recorded through Unity for the traditional Flanker Task and for the GA.

### 2.1. Flanker Task

Following task parameters and directions of the child-friendly Flanker Task [46], participants were told that the middle fish was hungry and they were instructed to feed the fish by pressing the button that matched the way the fish was pointing. The target array of fish appeared and remained on the screen until the child made a response, to a maximum of 1700 ms. The intertrial interval was 450 ms. Trials in which the child did not respond within 1700 ms (omission) or responded incorrectly (commission) were recorded as errors, and the next trial began. To follow the traditional Flanker Task design that provides auditory feedback on performance: correct responses cued an auditory positive chime and incorrect responses cued an auditory negative ratchet-like tone.

### 2.2. Gamified Assessment of Flanker

**Figure 1:**
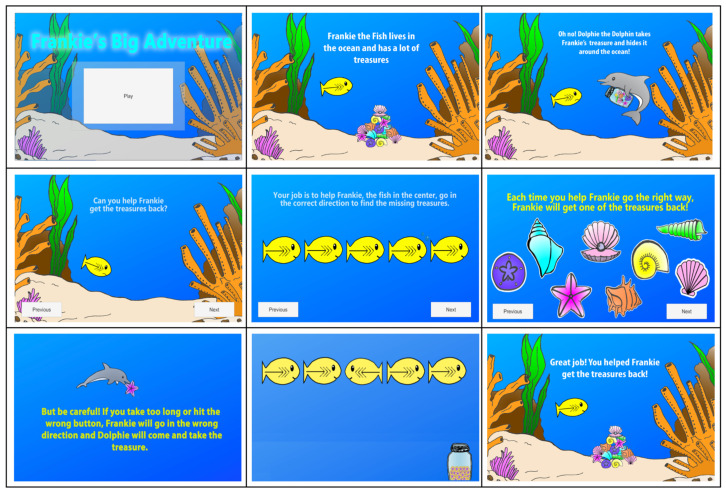
Gamified Flanker narrative, instructions, feedback, and progress. Gamification features were illustrated and developed by the first author.

The GA Condition applied gamification to the traditional Flanker Task, but the main goal of inhibiting distraction from the flanking fish and responding to the direction of the central target fish remained the same [59]. One feature from the traditional Flanker Task employed in the GA was auditory positive and negative feedback corresponding to correct and incorrect responses (described above in Section 2.1). The evidence-based game mechanics included a narrative, positive incentives, ambient linear music, anticipation of an opponent’s behavior, real-time feedback on performance progress, and a staircase algorithm to continuously adapt the difficulty level based on performance [55]. Many iterations of the narrative were hand-drawn, digitized, animated and then pilot tested. The final developmentally appropriate narrative presented to participants was for children to help Frankie, the center fish, go in the correct direction to recover ocean treasures (reward) from Dolphie the Dolphin (opponent). For providing correct responses, in which children helped Frankie go in the correct direction, participants received an ocean treasure as positive feedback (reward system). For each correct trial, an animation of the treasure would go into a jar and increase throughout the game, so children were able to continuously see visual progress of their performance throughout the game (feedback system). Because preschool children cannot fluently read yet, treasures increasing in a jar was found to be a developmentally appropriate visual feedback system for this age group during pilot testing, in contrast to feedback systems such as written objectives and leaderboard rankings employed with older populations. If children took too long to make a response or responded in the wrong direction, Dolphie the Dolphin would come and take a treasure as negative feedback (motivation; see Figure 1). 

The response time allotted for each trial was adjusted based on the individual capabilities of the children utilizing a machine learning algorithm that executed a staircase level structure, dynamic adjustment based on performance, and stagnation of difficulty. This algorithm ensured that the task was appropriately challenging for each participant, taking into account diverse cognitive profiles of young children. The 42 trials were divided into 14 hidden levels with each level consisting of three trials (hidden in the sense that participants were not explicitly shown the level structure, but the level structure is embedded into the game mechanics). These trials increased in difficulty gradually. If children responded correctly for three consecutive trials, the allotted response time decreased by 500 milliseconds (ms), making the task more challenging. If children responded incorrectly or took too long to respond, the difficulty level remained the same, and the allotted response time for that level did not change until children achieved three consecutive correct responses. This approach ensured that the GA adapted to the abilities of the children, providing an appropriately challenging and engaging experience while taking into account individual differences in EF. It also encouraged skill development by incrementally increasing the difficulty level as the children demonstrated proficiency.

### 2.3. Enjoyment and Preference Measures

Children were presented with a 5-point Smileyometer likert scale with five faces ranging from a frowny face (really disliked) to a big smiley face (really liked) at the end of each assessment to measure children’s enjoyment of each activity [60]. Additionally, children were administered a This or That survey instrument, a valid measure of children’s enjoyment of a technology experience through a relative comparison where children indicate which task (out of two) that they would like to play again [61,62].

### 2.4. Standardized Academic Achievement Measures

Children were administered two Wechsler Preschool and Primary Scale of Intelligence (WPPSI-P; [63]) Subtests as standardized instruments of Academic Achievement at subsequent lab visits. The Verbal Information Subtest was administered to assess verbal skills and the Matrix Reasoning Subtest was administered to assess mathematical skills for preschool-aged children. The Verbal Information subtest consists of 34 questions that assess knowledge of general acquired facts as a proxy for verbal intelligence. The Matrix Reasoning subtest consists of 29 problem sets in which children view an incomplete matrix followed by the selection of which option completes the matrix. These problem sets assess knowledge of part–whole relationships, perceptual organization, and classification and spatial capacity.

## 3. Results

First, we examined the conflict resolution induced by both Conditions, hypothesizing slower RT and lower accuracy on incongruent trials compared to congruent trials. Second, we examined the association between performance on the GA and the traditional Flanker Task. Then, we reported the associations between GA performance and standardized academic achievement outcomes known to be associated with performance on the traditional Flanker Task. Lastly, we compared children’s rating of enjoyment and preference between the GA and the Flanker Task.

### 3.1. Task Performance

**Figure 2:**
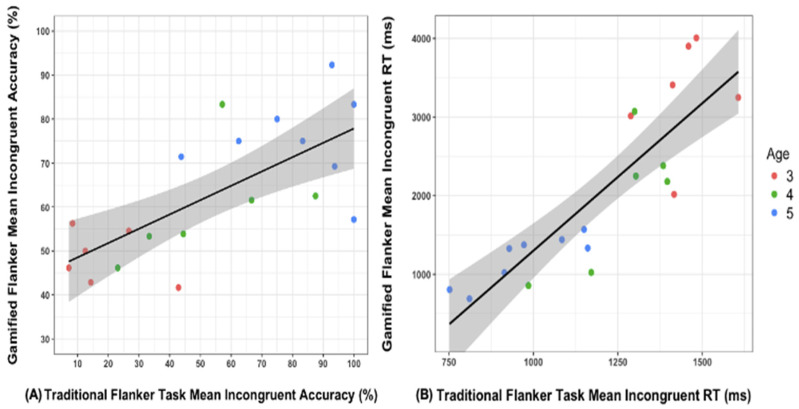
Gamified Assessment mean (**A**) accuracy and (**B**) reaction time on incongruent trials were correlated with performance on the traditional measure of executive function. Shaded regions represent the 95% confidence interval of the prediction line. Data points are displayed by the age bracket of participants to visualize developmental differences in performance. Note: RT = reaction time in milliseconds.

As expected, mean accuracy on congruent trials (*M* = 68.35%, *SD* = 26.35) was higher compared to mean accuracy on incongruent trials (*M* = 53.76%, *SD* = 32.46) in the Flanker Task Condition (paired-sample *t* = 4.82, *SE* = 3.02, 95%CI [8.26, 20.93], *p* < 0.001, Cohen’s *d* = 1.08). Mean accuracy on congruent trials (*M* = 88.43%, *SD* = 8.22) was higher compared to mean accuracy on incongruent trials (*M* = 62.78%, *SD* = 15.04), paired-sample *t* = 9.80, *SE* = 2.62, 95%CI [20.17, 31.12], *p* < 0.001, Cohen’s *d* = 2.19 in the GA Condition. The mean accuracy values are consistent with prior studies showing accuracy with children of similar age varying greatly from 22%–90% on incongruent trials for EF tasks [64]. There were no significant differences in accuracy on the Incongruent trials of the Flanker Task compared to the GA, paired-sample *t* = 1.66, *SE* = 5.45, 95%CI [2.38, 20.43], *p* = 0.114. There was a positive correlation between Conditions for the main dependent outcome variable of mean incongruent accuracy, *r* = 0.703, *p* < 0.001, 95%CI [ 0.377, 0.874] (see Figure 2A). Accuracy on the congruent trials (*r* = 0.731, *p* < 0.001) and overall accuracy (*r* = 0.789, *p* < 0.001) between Conditions were also positively correlated.

Mean RT on congruent trials (*M* = 1019.11 ms, *SD* = 193.38) was faster compared to mean RT on incongruent trials (*M* = 1198.75 ms, *SD* = 243.75) in the Flanker Task Condition (paired-sample *t* = 7.12, *SE* = 25.22, 95%CI [126.86, 232.43], *p* < 0.001, Cohen’s *d* = 1.59). Similar to the findings with accuracy, mean RT on congruent trials (*M* = 1656.45 ms, *SD* = 813.83) was faster than mean RT on incongruent trials (*M* = 2046.10 ms, *SD* = 1069.07) in the GA Condition, (paired-sample *t* = 5.50, *SE* = 70.89, 95%CI [241.27, 538.04], *p* < 0.001, Cohen’s *d* = 1.23). Furthermore, there was a positive and significant correlation in mean incongruent RT between Conditions, *r* = 0.855, *p* < 0.001 95%CI [0.664, 0.941] (see Figure 2B). These results of children showing significant differences between congruent and incongruent trial performance in both Conditions replicate the well-known conflict effect induced by the traditional Flanker Task and indicate that the GA similarly required children to resolve conflict and induced EF demands. Children performing better on congruent trials compared to incongruent trials required children to override the prepotent action to respond in the direction of the distractor fish during incongruent trials (inhibitory control), adapt responses when the target switched directions and when the task dimensions changed between congruent and incongruent trials (cognitive flexibility), and remembered and followed the task goal of paying attention to the central fish while simultaneously monitoring competing demands of ignoring the distracting fish (working memory).

### 3.2. Harnessing Machine Learning to Accommodate Diverse Developmental Profiles

Conflict Effect RT scores were computed by subtracting mean congruent RT from mean incongruent RT. Conflict Effect RT scores of the GA and Flanker Task were positively and significantly correlated, *r* = 0.635, *p* = 0.003 95%CI [0.268, 0.841]. An unexpected finding was that conflict RT scores of the GA (*M* = 389.65 ms, *SD* = 317.04) were larger compared to conflict RT scores of the traditional Flanker Task (*M* = 179.65 ms, *SD* = 112.78, paired-sample *t* = 3.61, *SE* = 58.22, 95%CI [88.14., 331.87], *p* = 0.002, Cohen’s *d* = 0.81). Conflict Effect accuracy scores were computed by subtracting mean incongruent accuracy from mean congruent accuracy. Conflict accuracy scores of the GA (*M* = 25.65%, *SD* = 11.71) were larger compared to conflict accuracy scores of the traditional Flanker Task (*M* = 14.59%, *SD* = 11.71, paired-sample *t* = 3.37, *SE* = 3.28, 95%CI [4.18., 17.93], *p* = 0.003, Cohen’s *d* = 0.75). These results showing conflict scores for both accuracy and RT indicate that the traditional and gamified Flanker Conditions taxed children’s EF.

Conflict Effect RT scores between the GA and Flanker Task were positively correlated, *r* = 0.635, *p* = 0.003, 95%CI [0.268, 0.841]. However, Conflict Effect accuracy scores between the GA and Flanker Task were not significantly correlated, *r* = 0.337, *p* = 0.147. Upon further inspection, it was found that while both the traditional Flanker Task and GA were more challenging for younger children compared to older children—a pattern consistent with prior literature—the fixed parameters of the traditional Flanker Task may have been too challenging for the youngest children. Traditional Flanker Task mean accuracy for 3-year-olds was much lower due to numerous *omission* errors, averaging 23 time-outs (SD = 4.10) for failing to respond within the allotted time, compared to 4.33 (SD = 4.08) and 1.13 (SD = 2.10), on average, for 4 and 5-year-olds, respectively. In the GA Condition, the machine learning adjusted the parameters to individual capabilities; thus, the 3-year-olds experienced fewer omission errors (M = 3.17, SD = 1.72). These results show that both the traditional Flanker Task and GA Condition produced conflict effects in RT and accuracy, but accuracy Conflict Effects between the Conditions were not correlated because the traditional Flanker Task resulted in many omission errors for the youngest children in the sample, skewing their overall accuracy toward the floor.

### 3.3. Association with Standardized Academic Achievement Measures

**Figure 3:**
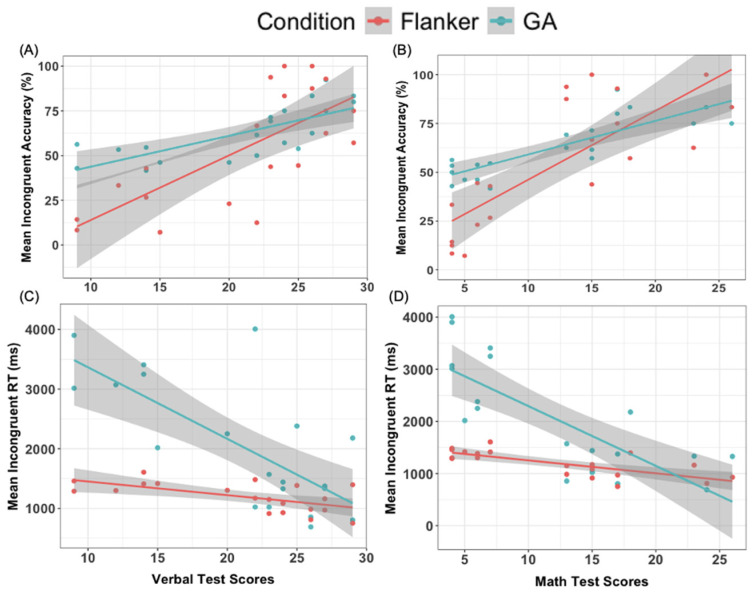
Scatterplots of Flanker Task and Gamified Assessment Performance with Standardized Wechsler Preschool and Primary Scale of Intelligence (WPPSI-P) Academic Achievement Scores. Positive associations were found between the mean Accuracy of both Conditions and (**A**) Verbal and (**B**) Math Scores. Negative associations were found between the mean Reaction Time of both Conditions and (**C**) Verbal (**D**) Math Scores. Shaded regions represent the 95% confidence interval of the prediction line.

Mean accuracy on the incongruent trials on the Flanker Task (*r* = 0.722, *p* < 0.001) and the GA (*r* = 0.744, *p* < 0.001) were positively associated with children’s WPPSI-P Verbal Subtest scores (see Figure 3A). Mean accuracy on the incongruent trials on the Flanker Task, (*r* = 0.790, *p* < 0.001) and the GA (*r* = 0.832, *p* < 0.001), were positively associated with children’s WPPSI-P Matrix Reasoning Subtest scores (see Figure 3B). In other words, children who had higher accuracy scores on the incongruent trials in both Conditions had higher math and verbal scores. Slower mean RT on the incongruent trials for the Flanker Task, (*r* = −0.609, *p* = 0.004) and the GA (*r* = −0.727, *p* < 0.001) were negatively associated with children’s WPPSI-P Verbal Subtest scores (see Figure 3C). Mean RT on the incongruent trials on the Flanker Task (*r* = −0.738, *p* < 0.001) and the GA (*r* = −0.779, *p* < 0.001), were negatively associated with children’s WPPSI-P Matrix Reasoning Subtest scores (see Figure 3D): children with faster average RT on the incongruent trials in both Conditions had higher math and verbal scores. These results indicate that GA performance was associated with standardized academic achievement outcomes known to be associated with performance on the traditional Flanker Task.

### 3.4. Gamified Assessment and Enjoyment

**Figure 4:**
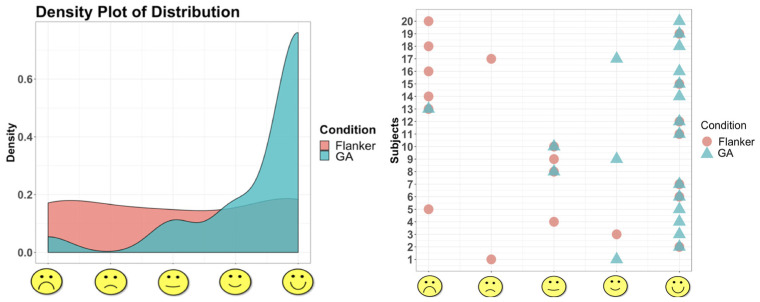
The density plot displays how children rated the gamified assessment as more enjoyable than the traditional Flanker Task. Density plots use kernel smoothing to estimate a real valued function as the weighted average of neighboring observed data [65]. The dot plot displays individual differences of enjoyment for the traditional Flanker and Gamified Conditions.

Enjoyment was measured on a 5-point Smileyometer likert scale. To assess possible order effects, a mixed factorial analysis of variance (ANOVA) was conducted on enjoyment, factoring order as a between-subject variable and Condition as the within-subject variable. There was a main effect of Condition, in that children’s enjoyment ratings were higher in the GA Condition (*M* = 4.45, *SD* = 1.05) compared to the Flanker Task Condition (*M* = 3.05, *SD* = 1.70), *F*(1, 18) = 13.67; *p* = 0.002; *ηp*2 = 0.43. There was no main effect of order, *F*(1, 18) = 0.34, *p* = 0.57. These results indicate that children’s enjoyment ratings of the GA were higher compared to the traditional Flanker Task, regardless of the order in which the tasks were played (see Figure 4).

### 3.5. Preference for the Gamified Assessment

**Figure 5:**
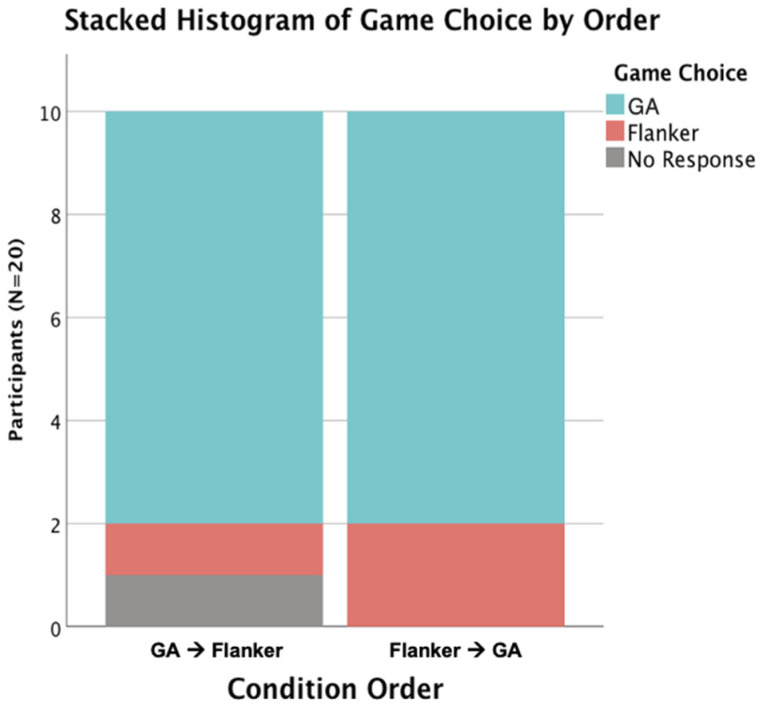
Regardless of order, 80% of the children preferred the gamified executive function task over the traditional executive function task.

Children selected the GA more than the Flanker Task as their free-choice game option when asked which game they would play again: 80% of children (16 out of 20) selected the GA while 15% of children (3 out of 20) selected the Flanker Task, and only one participant did not respond. A chi-square test of independence was performed to examine the relation between choice and task order (GA-then-Flanker vs. Flanker-then-GA). The relation between these variables was not significant, *χ*^2^(2, *N* = 20) = 1.33, *p* = 0.51, indicating that regardless of which order children played the tasks, the choice to play the GA over the Flanker Task was chosen by the majority of children (see Figure 5).

## 4. Discussion

The goal of this pilot study was to investigate the feasibility of a GA of EF with preschool children. Children’s performance on the GA was associated with performance on the traditional EF measure. The preliminary results suggest that with the incorporation of gamification, the assessment still produced the well-established conflict effect the Flanker Task induces: children demonstrated slower RT and lower accuracy on incongruent trials compared to congruent trials. Performance on the gamified Flanker Task was associated with performance on standardized mathematical and verbal academic achievement assessments. Aligned with prior research [46], task performance was sensitive to age: the trend in the data displayed younger children exhibiting slower RT, lower accuracy, and more omission errors compared to older children.

These preliminary findings propose that integrating evidence-based gamification into a conventional assessment of EF did not hinder children’s performance and is a useful method to provide insight into individual differences of EF. Children displayed a greater preference for and rated their enjoyment of the GA higher compared to the traditional version of the Flanker Task. An initial pattern observed from this feasibility study is that these methods may be particularly beneficial for younger children, such as 3-year-olds in preventing floor effects. The adaptability to real-time performance that machine learning permits in EF measures may be advantageous to ensure that the task is challenging individuals at the right level. GAs of EF show potential for diverse age and cognitive profiles and harness what we know about human drive for curiosity, incentives, competition, and fun. These preliminary findings present initial results that GAs can be employed as ecologically-relevant and engaging assessments of EF in preschool populations.

While the GA of EF with machine learning incorporated into a traditional measure of EF yielded associations with performance on the traditional measure of EF, math and verbal skill performance, and children exhibited increased enjoyment, there are limitations that warrant future research on this topic. This was a feasibility study with a limited sample size, and more participants are required to evaluate the robustness and replicability of these findings. Examining associations between children’s performance on the GA with teacher and adult reports of EF in classroom and home contexts would provide valuable insight on the ecological validity of the assessment. Studies on individualized assessments with adults have shown that tasks developed to adapt based on individual performance from real-time data have been shown to have higher prediction accuracy of performance and account for variations across individuals compared to non-individualized approaches [66]. More recent studies with adults following this dynamic approach have found that the data are normally distributed and more sensitive to assessing differences in EF within a general population [67]. Whether this individualized approach would have the same benefits for preschool children is an open question for future investigation.

To investigate changes in task performance over time and a more detailed microanalysis of the effects stemming from the game mechanics, it is essential to incorporate learning curve and trial-by-trial data. Integrating learning curve analyses would also clarify the stage(s) when children start to improve and eventually plateau with additional practice on both tasks, if at all [68]. Following the promising initial results of this preliminary experiment, we developed a function to generate learning curve data for subsequent studies. Investigating GAs with more trials and extended testing durations would clarify the efficacy of integrating gamification into traditional tasks on both children’s performance and engagement (specifically, when it begins to dwindle) over longer periods of time. Addressing these limitations in future research presents endeavors to investigate whether GAs can not only provide a potential solution to the current individual difference challenges of employing EF assessments in the preschool period when EF is rapidly developing.

Computerized GAs can permit greater accessibility for individuals who may have constraints attending in-person sessions physically or geographically, enable standardized administration to decrease implicit human bias, allow for automatized scoring and data entry to reduce the load on researchers and the risk of error from manual data entry, adapt for dynamic adjustment of difficulty levels based on individual performance to prevent floor or ceiling effects on performance, increase enjoyment and motivation to reduce participant disengagement, and pave routes for many future research endeavors for both EF assessment and interventions [69]. While there are advantages to digitizing EF assessments, there are also disadvantages that should be noted such as digital literacy, access to technology, and privacy concerns. Thus, when employing GAs, the specific needs of the population, the goals of the assessment, and the context should be taken into account.

## 5. Conclusions

This within-subjects experiment conducted in a carefully controlled laboratory setting with children ages 3–5 assessed the feasibility of incorporating theory-driven gamification and machine learning into a traditional EF assessment. The preliminary results indicate that the GA did not change essential task properties and the well-established conflict effect induced by the Flanker Task was exhibited in both accuracy and reaction time outcomes of the GA. The GA showed a similar pattern of correlation with verbal and math scores compared to the traditional EF assessment. The GA did not hinder the ability to measure individual differences in EF and children rated the GA as enjoyable. These pilot findings indicate that this line of work holds promise to be implemented with a more powered sample on a larger scale to validate the construct and criterion validity of the GA. Adaptive, enjoyable, and ecologically relevant computerized GAs may offer a novel approach to mitigate floor or ceiling effects with preschool children and serve as a cost-effective and scalable method for inclusivity across diverse age and cognitive profiles. Digitized EF assessments with the incorporation of machine learning that adapts to individual profiles represent one potential solution to streamline the laborious process of developing additional task conditions with manual scoring to adjust difficulty levels. While GAs should not replace traditional childhood EF assessments, which are fundamental to our understanding of cognitive development, well-developed GAs—built upon evidence-based and intentional practices—can complement established EF assessments. This supplementation can contribute to a more comprehensive assessment of childhood EF in the digital era.

## Figures and Tables

**Table 1:** Summary of Flanker Task versus Gamified Assessment Features.

Task Features	Flanker	Gamified Flanker
Practice Trials	8	8
Inter-trial Duration	450 ms	450 ms
Auditory Feedback	✓	✓
Linear Music	X	✓
Number of Trials	42	42
Narrative	X	✓
Visual Feedback	X	Rewards & Competitor
Player Adaptability/Trial Duration	1700 ms (fixed)	Incremental Challenge ^1^

^1^ Allotted response time dynamically adjusted based on performance in incremental challenge of 500 milliseconds (ms) using a machine learning staircase algorithm.

## Data Availability

The data presented in this study are available on request from the corresponding author because the materials and methods utilized in this study, including the gamified assessment developed, are not publicly available as they have been transformed into an executive function (EF) training paradigm for an ongoing larger-scale study. Data from the current study will be made available upon request. Once the larger umbrella study resulting from this feasibility study is published, the materials, methods, and data codebook will be accessible on the Open Science Framework.

## Supplementary Materials

The following supporting information can be downloaded at: https://www.mdpi.com/article/10.3390/brainsci14050451/s1, Video S1: Example Clip of Gamified Flanker Sub A. Video S2: Example Clip of Traditional Flanker Sub A. Video S3: Example Clip of Gamified Flanker Sub B. Video S4: Example Clip of Traditional Flanker Sub B.
